# Disruption of *ABI4* Enhances Anthocyanin Accumulation in *Arabidopsis* Seedlings Through HY5-Mediated Light Signaling

**DOI:** 10.3390/plants14131905

**Published:** 2025-06-20

**Authors:** Mingyang Zeng, Yan Wu, Shunfa Lin, Fang Zhang, Haiyan Jiang, Lixia Ma, Dong Liu

**Affiliations:** Ministry of Education Key Laboratory of Crop Physiology, Ecology and Genetic Breeding, Jiangxi Agricultural University, Nanchang 330045, China; myzengbio@163.com (M.Z.); wuyanbio@163.com (Y.W.); jxaulin999@163.com (S.L.); zhangf0124@126.com (F.Z.); haiyan200228@163.com (H.J.); malxjxau@126.com (L.M.)

**Keywords:** *Arabidopsis thaliana*, anthocyanin, ABI4, HY5, light signaling

## Abstract

The AP2/ERF transcription factor ABSCISIC ACID INSENSITIVE 4 (ABI4) plays diverse roles in plant development and responses to abiotic stress. However, its potential involvement in regulating anthocyanin biosynthesis is not fully understood. In this study, three different loss-of-function *abi4* alleles (*abi4-1*, *abi4-2*, and *abi4-101*) were employed to investigate the role of ABI4 in the regulation of anthocyanin accumulation in *Arabidopsis* seedlings. These *abi4* mutants exhibited significantly increased anthocyanin accumulation, which was associated with elevated expression of genes involved in anthocyanin biosynthesis. HY5 (LONG HYPOCOTYL 5), a central component of photomorphogenesis, acts as a key light-regulated molecular switch. Further analysis revealed that ABI4 requires HY5 to function as a negative regulator of anthocyanin biosynthesis. Additionally, loss of *ABI4* resulted in heightened light sensitivity, leading to increased light-induced chlorophyll accumulation and chloroplast development, along with upregulation of photosynthesis-related genes. Interestingly, the light-hypersensitive phenotype of *abi4* mutants was partially rescued by the loss of HY5 function. Taken together, these findings demonstrate that *ABI4* negatively regulates anthocyanin accumulation in *Arabidopsis* seedlings through a HY5-dependent light signaling pathway.

## 1. Introduction

Anthocyanins are a large group of plant secondary metabolites classified as flavonoids, playing essential roles in various physiological and biological processes in vascular plants. For example, anthocyanins present in vegetative tissues help protect plants from excessive light and UV radiation, while their accumulation in reproductive organs attracts pollinators and animals, facilitating pollination and seed dispersal [1,2].

The biosynthetic pathway of anthocyanins in vascular plants is well established. In the model plant *Arabidopsis thaliana*, anthocyanin biosynthesis is genetically regulated by two classes of genes: early biosynthetic genes (EBGs) and late biosynthetic genes (LBGs) [3,4]. The EBGs including *CHS* (*CHALCONE SYNTHASE*), *CHI* (*CHALCONE ISOMERASE*), *F3H* (*FLAVANONE 3*-*HYDROXYLASE*), and *F3′H* (*FLAVONOID 3-HYDROXYLASE*) participate in the common steps of the flavonoid pathway. The LBGs, such as *DFR* (*DIHYDROFLAVONOL 4-REDUCTASE*), *LDOX* (*LEUCOANTHOCYANIDIN OXYGENASE*), *ANR* (*ANTHOCYANIDIN REDUCTASE*), and *UF3GT* (*UDP-GLUCOSE: FLAVONOID 3-O-GLUCOSYLTRANSFERASE*), function in the later stages of anthocyanin biosynthesis. EBGs are primarily regulated by at least three R2R3-MYB transcription factors: MYB11, MYB12, and MYB111. In contrast, LBGs are typically regulated by the well-known MYB-bHLH-WD40 (MBW) complex [5].

Anthocyanin biosynthesis is influenced by multiple environmental and endogenous cues. Among these, light is undoubtedly the most critical factor, although other environmental stresses such as low temperature, drought, nutrient (nitrogen and phosphorus) deficiency, wounding, and pathogen infection also contribute [6]. Notably, studies have shown that the application of exogenous sugars and hormones does not induce anthocyanin biosynthesis in the absence of light, indicating that light is a prerequisite for anthocyanin accumulation [7,8]. In *Arabidopsis*, light signals are perceived and mediated by several types of photoreceptors, including cryptochromes, phototropins, phytochromes, and UV RESISTANCE LOCUS 8 (UVR8) [9]. Numerous downstream components of these photoreceptors have been identified, including COP1 (CONSTITUTIVE PHOTOMORPHOGENIC 1) and HY5 (LONG HYPOCOTYL 5). These two key regulators act antagonistically in controlling light-mediated seedling development. The bZIP transcription factor HY5 serves as a positive regulator of photomorphogenesis, whereas COP1 negatively regulates *HY5* [10,11]. Mutations in *HY5* result in a long-hypocotyl phenotype under all light conditions, suggesting that HY5 functions downstream of all photoreceptors to promote photomorphogenesis [9,12]. Beyond its central role in light signaling, HY5 also regulates the transcription of anthocyanin biosynthetic genes by directly binding to the promoters of both EBGs and LBGs [13]. In addition, HY5 modulates the expression of other transcription factors, thereby co-regulating downstream structural genes and influencing anthocyanin accumulation in *Arabidopsis* seedlings. For instance, transcription factors such as PAP1 (PRODUCTION OF ANTHOCYANIN PIGMENT 1), MYBD (MYB-LIKE DOMAIN), and MYBL2 (MYB-LIKE 2) are transcriptionally regulated in response to light and act downstream of HY5, functioning as either positive or negative regulators of anthocyanin biosynthesis [14,15,16].

The phytohormone abscisic acid (ABA) plays multiple roles in regulating developmental and physiological processes, including seed maturation and germination, stomatal closure, and stress adaptation [17,18]. ABI4 is a member of the plant-specific AP2/ERF transcription factor family and was initially identified as an important positive regulator in the ABA signaling cascade. Subsequent studies have shown that ABI4 is a multifunctional regulatory factor involved in lateral root development, lipid metabolism, plastid-to-nucleus retrograde signaling, and abiotic stress responses [19,20,21]. ABA biosynthesis mutants such as *aba1*, *aba2*, and *aba3* exhibit reduced tolerance to drought and high-salt stress due to impaired ABA production [22]. In contrast, the ABA signaling mutant *abi4* displays enhanced resistance to drought, salt, and osmotic stresses [23,24,25,26]. Anthocyanins are important for protecting plants from oxidative damage caused by environmental stressors [27,28]. While the role of ABI4 in abiotic stress responses is well documented, its potential involvement in regulating anthocyanin biosynthesis is not fully understood. In the present study, it was found that disruption of *ABI4* leads to enhanced anthocyanin accumulation in *Arabidopsis* seedlings. Further investigation revealed that HY5 is required for ABI4’s function in regulating anthocyanin biosynthesis.

## 2. Results

### 2.1. Loss-of-Function Mutations in ABI4 Enhances Anthocyanin Accumulation in Arabidopsis Seedlings

**Figure 1:**
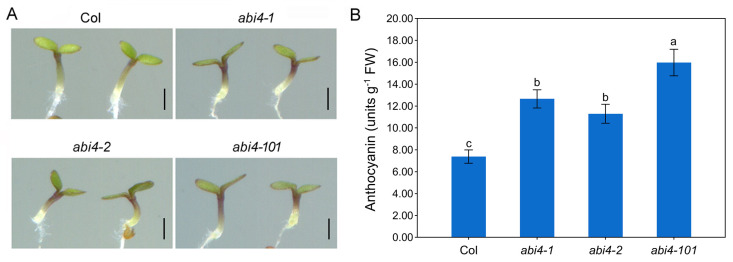
Anthocyanin accumulation in wild-type, *abi4*-*1*, *abi4*-*2*, and *abi4*-*101* mutants. (**A**) Representative phenotypes of wild-;-type (Col) and *abi4* mutant seedlings (*abi4*-*1*, *abi4*-*2*, and *abi4*-*101*) grown on 1/2 MS medium for 4 days. Scale bar = 1 mm. (**B**) Anthocyanin content in wild-type (Col) and *abi4* mutants (*abi4*-*1*, *abi4*-*2*, and *abi4*-*101*) grown on 1/2 MS medium for 4 days. One-way analysis of variance (ANOVA) with Duncan’s test was used to obtain significant variations. The different lowercase letters indicate significant differences among the samples at *p* < 0.05, *n* = 3.

To investigate whether ABI4 is involved in modulating anthocyanin biosynthesis in *Arabidopsis*, seeds of three loss-of-function *abi4* mutant alleles (*abi4-1*, *abi4-2*, and *abi4-101*) and their corresponding wild-type (Col) were sown and grown on 1/2 MS solid medium supplemented with 2% sucrose for 4 days. Phenotypic analysis showed that the localization of accumulated anthocyanins in *abi4* mutant seedlings was similar to that of the wild type. Anthocyanin pigmentation was primarily observed at the edges of cotyledons and more prominently in the upper part of hypocotyls (Figure 1A). Although disruption of *ABI4* did not alter the spatial pattern of anthocyanin accumulation, the levels of anthocyanin were clearly elevated in the *abi4* mutants, particularly at the cotyledon margins and the upper hypocotyl, compared to wild-type seedlings (Figure 1A). Quantitative analysis further confirmed that anthocyanin content was significantly higher in the *abi4* mutants than in the wild type, consistent with visual observations (Figure 1B).

### 2.2. Light Is Essential for ABI4 Function in Regulating Anthocyanin Biosynthesis

**Figure 2:**
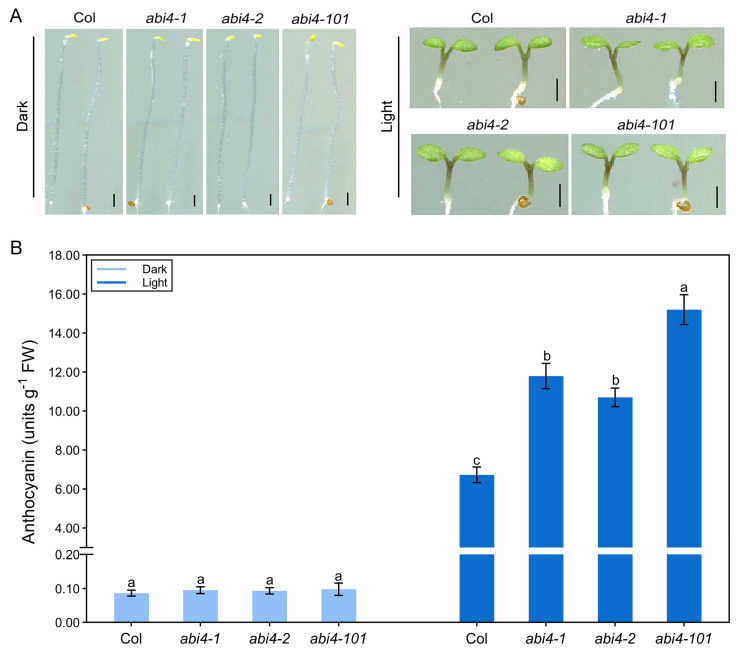
Anthocyanin levels in wild-type and *abi4* mutant seedlings grown under dark and light conditions. (**A**) Representative phenotypes of wild-type (Col) and *abi4* mutants (*abi4*-*1*, *abi4*-*2*, and *abi4*-*101*) grown under dark or light conditions for 4 days. Scale bar = 1 mm. (**B**) Anthocyanin content in wild-type (Col) and *abi4* mutants grown under dark or light conditions for 4 days. One-way analysis of variance (ANOVA) with Duncan’s test was used to obtain significant variations. The different lowercase letters indicate significant differences among the samples at *p* < 0.05, *n* = 3.

Light is a key environmental cue regulating anthocyanin accumulation in vascular plants. To determine whether *ABI4*’s role in regulating anthocyanin biosynthesis depends on light, we compared anthocyanin levels in wild-type and *abi4* mutant seedlings grown under dark and light conditions. Under dark conditions, both wild-type and *abi4* seedlings accumulated only minimal anthocyanin, with no significant differences observed among genotypes (Figure 2). However, under light conditions, anthocyanin accumulation was markedly increased in *abi4* mutants compared to the wild type (Figure 2). These findings suggest that light has an important effect on anthocyanin accumulation in *abi4* mutant seedlings.

### 2.3. ABI4 Requires the Presence of HY5 to Negatively Regulate Anthocyanin Biosynthesis

**Figure 3:**
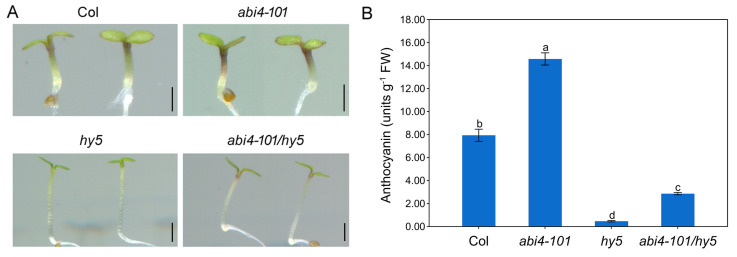
Anthocyanin accumulation in wild-type, single mutants of *abi4*-*101* and *hy5*, and the *abi4*-*101*/*hy5* double mutant. (**A**) Representative phenotypes of wild-type (Col), single mutants (*abi4*-*101* and *hy5*), and the double mutant (*abi4*-*101*/*hy5*) grown on 1/2 MS medium for 4 days. Scale bar = 1 mm. (**B**) Anthocyanin content in wild-type (Col), single mutants (*abi4*-*101* and *hy5*), and the double mutant (*abi4*-*101*/*hy5*) grown on 1/2 MS medium for 4 days. One-way analysis of variance (ANOVA) with Duncan’s test was used to obtain significant variations. The different lowercase letters indicate significant differences among the samples at *p* < 0.05, *n* = 3.

The above results indicated that loss of *ABI4* function promotes anthocyanin accumulation in a light-dependent manner. HY5 (LONG HYPOCOTYL 5) is a core regulator of photomorphogenesis and acts as a light-responsive developmental switch. Previous studies have also shown that HY5 promotes anthocyanin biosynthesis by activating both structural genes and transcription factors involved in the pathway [13,14,15]. Based on this, we hypothesized that HY5 may be required for ABI4-mediated repression of anthocyanin biosynthesis. To test this, the double mutant *abi4*-*101*/*hy5* was generated by crossing *abi4*-*101* with *hy5*, and double homozygous lines were selected from the F_2_ generation. Anthocyanin levels were then measured in wild-type, single mutants (*abi4*-*101* and *hy5*), and the *abi4*-*101*/*hy5* double mutant. As expected, *abi4*-*101* showed significantly higher anthocyanin levels compared to the wild type, while the *hy5* mutant had much lower levels (Figure 3). However, the *abi4*-*101*/*hy5* double mutant did not exhibit enhanced anthocyanin accumulation compared to the wild-type or the *abi4*-*101* single mutant (Figure 3). These findings indicate that ABI4 requires HY5 to act as a negative regulator of anthocyanin biosynthesis.

### 2.4. ABI4-Mediated Negative Regulation of Photosynthetic Development Requires Functional HY5

**Figure 4:**
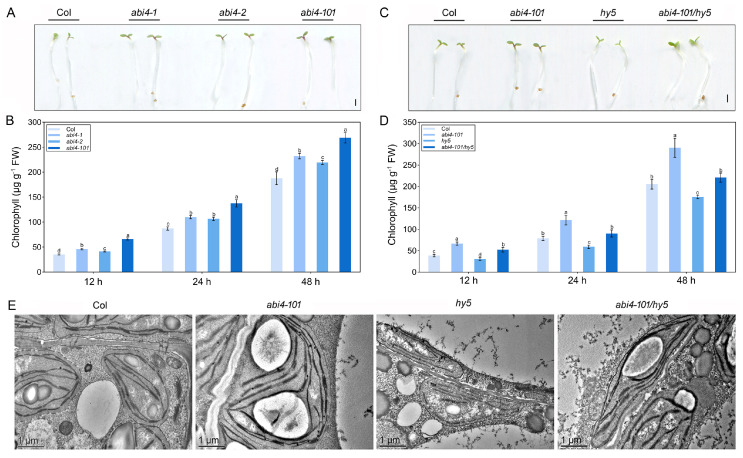
Photosynthetic development of wild-type, single mutants of *abi4*-*101* and *hy5*, and the *abi4*-*101*/*hy5* double mutant. (**A**) Representative phenotypes and (**B**) chlorophyll content of wild-type (Col) and *abi4* mutants (*abi4*-*1*, *abi4*-*2*, and *abi4*-*101*) grown in darkness for 4 days followed by continuous light exposure for 12–48 h. (**C**) Representative phenotypes, (**D**) chlorophyll content, and (**E**) chloroplast ultrastructure of wild-type (Col), single mutants (*abi4*-*101* and *hy5*), and the double mutant (*abi4*-*101*/*hy5*) grown for 4 days in darkness followed by continuous expose to light for 12–48 h. One-way analysis of variance (ANOVA) with Duncan’s test was used to obtain significant variations. The different lowercase letters indicate significant differences among the samples at *p* < 0.05, *n* = 3.

Given that light is essential for *ABI4* function, we next examined whether loss of *ABI4* affects seedling sensitivity to light during early development. Since light promotes chlorophyll biosynthesis and chloroplast development during de-etiolation, we compared responses to light in etiolated wild-type and *abi4* mutant seedlings. As shown in Figure 4A, cotyledons of *abi4* mutant seedlings appeared slightly greener than those of wild-type seedlings after light exposure. Quantitative analysis confirmed that chlorophyll content was significantly higher in *abi4* mutants compared to the wild type during de-etiolation (Figure 4B). In addition, transmission electron microscopy (TEM) analysis revealed that chloroplasts in *abi4-101* had larger size, more thylakoids, and larger starch granules compared to wild-type chloroplasts (Figure 4E).

To determine whether HY5 is required for ABI4-mediated regulation of photosynthetic development, we analyzed chlorophyll content and chloroplast structure in wild-type, *abi4*-*101*, *hy5*, and *abi4*-*101*/*hy5* seedlings. Following light exposure, *abi4*-*101* seedlings showed dark-green cotyledons, while *hy5* seedlings remained pale green. The *abi4*-*101*/*hy5* double mutant had an intermediate phenotype, with greener cotyledons than *hy5* but less green than *abi4*-*101* (Figure 4C). Chlorophyll quantification showed that *abi4*-*101* accumulated more chlorophyll than the wild type, while *hy5* had less. The *abi4*-*101*/*hy5* double mutant had chlorophyll levels lower than *abi4*-*101* but higher than *hy5* (Figure 4D). Ultrastructural analysis of chloroplasts confirmed that *hy5* seedlings had smaller chloroplasts with fewer thylakoids and smaller starch granules. While *abi4*-*101* had well-developed thylakoid structures, the *abi4*-*101*/*hy5* double mutant showed less developed thylakoids and fewer grana stacks (Figure 4E). These results collectively indicate that ABI4 negatively regulates the light-induced development of the photosynthetic apparatus, and this function is at least partially dependent on HY5.

### 2.5. Disruption of ABI4 Alters the Expression of Genes Involved in Anthocyanin Biosynthesis and Photosynthesis

**Figure 5:**
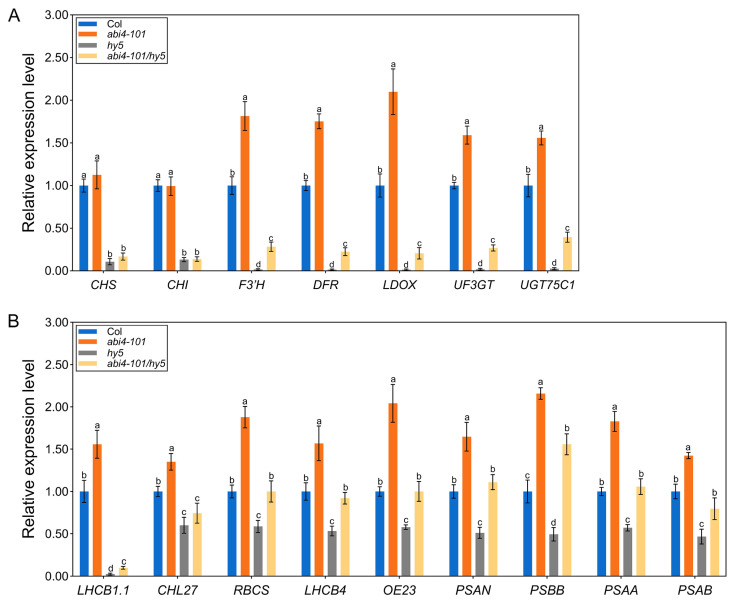
Expression levels of the key genes involved in anthocyanin biosynthesis and photosynthesis. (**A**) Transcript levels of anthocyanin biosynthetic structural genes in wild-type (Col), single mutants (*abi4*-*101* and *hy5*), and the double mutant (*abi4*-*101*/*hy5*). (**B**) Transcript levels of photosynthesis-related genes in the wild-type (Col), single mutants (*abi4*-*101* and *hy5*), and double mutant (*abi4*-*101*/*hy5*). One-way analysis of variance (ANOVA) with Duncan’s test was used to obtain significant variations. The different lowercase letters indicate significant differences among the samples at *p* < 0.05, *n* = 3.

To explore the molecular basis for increased anthocyanin accumulation in *abi4* mutants, we analyzed transcript levels of anthocyanin biosynthetic genes. These included early biosynthetic genes (EBGs: *CHS*, *CHI*, *F3’H*) and late biosynthetic genes (LBGs: *DFR*, *LDOX*, *UF3GT*, and *UGT75C1*). As shown in Figure 5A, the expression levels of *F3′H*, *DFR*, *LDOX*, *UF3GT*, and *UGT75C1* were significantly upregulated in *abi4-101* compared to the wild type. However, *CHS* and *CHI* levels remained unchanged. These results suggest that ABI4 mainly suppresses anthocyanin biosynthesis by repressing LBGs. In contrast, both EBGs and LBGs were significantly downregulated in the *hy5* mutant, consistent with previous reports [13,14]. Furthermore, expression of all anthocyanin pathway genes was substantially lower in the *abi4-101*/*hy5* double mutant than in the *abi4-101* single mutant (Figure 5A).

We also examined the expression of photosynthesis-related genes, including the genes that encode light harvesting chlorophyll A/B-binding protein1.1 (LHCB1.1), CHLH subunit of Mg chelatase (CHL27), a member of the rubisco small subunit (RBCS), light harvesting chlorophyll A/B-binding protein4 (LHCB4), oxygen-evolving complex 23 (OE23), a subunit of photosystem I (PSAN), photosystem II reaction center protein B (PSBB), photosystem I reaction center protein PSAA (PSAA), and D1 subunit of the photosystem I reaction center (PSAB). These genes were all more highly expressed in *abi4*-*101* than in the wild-type and less expressed in *hy5* mutant (Figure 5B). In the *abi4*-*101*/*hy5* double mutant, expression levels of these genes were significantly reduced compared to *abi4*-*101*, though most remained higher than in *hy5* mutant. The exception was *CHL27*, whose expression was similar between *abi4*-*101*/*hy5* and *hy5* (Figure 5B). These findings suggest that ABI4 and HY5 have antagonistic roles in regulating photosynthesis-related gene expression in *Arabidopsis* seedlings.

## 3. Discussion

ABI4 is a member of the AP2/ERF transcription factor family, originally identified as a key component of the ABA signaling pathway [19,21]. Over the past few decades, accumulating evidence has revealed that ABI4 is a multifunctional regulatory factor involved in various biological processes [20]. However, its potential involvement in regulating anthocyanin biosynthesis is not fully understood. This study aimed to investigate the function of ABI4 in the biosynthesis of anthocyanins in *Arabidopsis* seedlings. Using three previously identified loss-of-function *abi4* mutants [25,29,30], we found that mutations in the *ABI4* gene led to significantly increased anthocyanin accumulation in seedlings (Figure 1). Further gene expression analysis revealed that disruption of *ABI4* enhanced the expression of late biosynthetic genes (LBGs; *DFR*, *LDOX*, *UF3GT*, and *UGT75C1*), but not early biosynthetic genes (EBGs; *CHS* and *CHI*; Figure 5A). These findings suggest that the disruption of *ABI4* enhanced anthocyanin accumulation in *Arabidopsis* seedlings primarily through the upregulation of expression of LBGs. Interestingly, a previous study reported that transgenic *Arabidopsis* lines overexpressing *MtABI4* showed decreased anthocyanin levels and enhanced cold tolerance [31]. Taken together, these findings suggest that ABI4 has significant negative effects on anthocyanin biosynthesis in *Arabidopsis* seedlings.

ABI4 regulates numerous aspects of plant growth and development in an ABA-dependent manner [20]. For example, exogenous ABA inhibits seed germination in wild-type *Arabidopsis*, whereas *abi4* mutants show enhanced germination, indicating that ABI4 mediates ABA-induced inhibition of seed germination [21]. Previous studies have also shown that ABA promotes anthocyanin accumulation in seedlings [32,33]. Considering that all three *abi4* mutant alleles are ABA-insensitive, and that our results show enhanced anthocyanin accumulation in these mutants (Figure 1), it is plausible that ABI4 regulates anthocyanin biosynthesis in an ABA-independent manner. Despite this, further studies will be needed to verify this hypothesis.

Light is a critical environmental factor that regulates anthocyanin biosynthesis, chloroplast development, and chlorophyll production. When dark-grown seedlings are exposed to light, etioplasts differentiate into mature chloroplasts, accompanied by the accumulation of photosynthetic pigments such as chlorophylls and carotenoids [34]. Given that ABI4 loss-of-function promotes anthocyanin biosynthesis in a light-dependent manner (Figure 2), we hypothesized that mutations in *ABI4* may alter the response of seedlings to light-induced photosynthetic development. Several lines of evidence support this hypothesis. First, *abi4* mutants accumulated more chlorophyll than the wild type when etiolated seedlings were transferred to light (Figure 4A,B). Second, disruption of *ABI4* enhanced chloroplast development and upregulated photosynthesis-related genes (Figure 4E and Figure 5B). Third, we found that ABI4-mediated negative regulation of photosynthetic development requires functional HY5 (Figure 3). Light is a key environmental signal controlling cotyledon greening and seedling de-etiolation [34]. Key regulators such as COP1 and HY5 are involved in this process. While HY5 promotes de-etiolation, COP1 targets HY5 for degradation via the 26S proteasome, inhibiting photomorphogenesis [10,35]. In our study, *hy5* mutant accumulated chlorophyll more slowly than the wild type upon light exposure, whereas *abi4-101* mutants de-etiolated faster (Figure 4C,D). Interestingly, the enhanced de-etiolation of *abi4-101* could be partially suppressed by HY5 loss (Figure 4C,D), suggesting that ABI4 and HY5 antagonistically regulate light-induced photosynthetic development in *Arabidopsis*.

As light is a primary regulator of anthocyanin biosynthesis, we investigated the effects of *ABI4* mutation under both dark and light conditions. No significant differences in anthocyanin levels were observed between wild-type and *abi4* mutants in the dark. However, under light, *abi4* mutants accumulated much more anthocyanin than their wild-type counterparts (Figure 2). This confirms that the altered anthocyanin biosynthesis in *abi4* mutants is light-dependent. The transcription factor HY5 acts downstream of multiple photoreceptors and plays a pivotal role in promoting photomorphogenesis [9,12]. In addition to regulating photomorphogenesis, it also regulates anthocyanin biosynthesis, nitrogen uptake, and abiotic stress responses [14,36,37,38]. It has been reported that HY5 plays a vital role in the biosynthesis of anthocyanin in various organs of plants such as leaves, stems, flowers and fruits [14,39,40,41]. In *Arabidopsis*, HY5 promotes anthocyanin biosynthesis by directly activating the expression of both EBGs and LBGs, and by inducing transcription factors involved in the pathway [13,14]. Given HY5’s central role in light signaling, we hypothesized that it may be involved in ABI4-regulated anthocyanin biosynthesis. Supporting this, we found that HY5 is required for the enhanced anthocyanin accumulation in *abi4-101* seedlings, as *abi4-101*/*hy5* double mutants exhibited dramatically lower anthocyanin levels compared to *abi4-101* single mutants (Figure 3). Furthermore, qRT-PCR analysis showed significantly reduced expression of anthocyanin biosynthetic structural genes in the double mutant (Figure 5A). These findings suggest that ABI4 regulates anthocyanin biosynthesis through HY5-mediated light signaling. Notably, the *hy5* mutation did not completely restore anthocyanin levels in the *abi4-101* background to those of *hy5* single mutants, indicating that ABI4 and HY5 antagonistically regulate this pathway.

## 4. Materials and Methods

### 4.1. Plant Materials and Growth Conditions

The wild-type *Arabidopsis thaliana* ecotype Columbia (Col) was used. The *abi4* mutant alleles (*abi4-1*: CS8104; *abi4-2*: SALK_080095; and *abi4-101*: CS3836) and *hy5* (SALK_096651C), all derived from the Col background, were obtained from the Arabidopsis Biological Resource Center. The *abi4-101*/*hy5* double mutant was generated by crossing *abi4-101* with *hy5*. The seeds were surface-sterilized by treatment with 75% ethanol for 30 s, followed by 1% NaClO for 10 min, and rinsed several times with sterile water. After cold stratification for 3 days at 4 °C, the seeds were germinated on 1/2 MS solid medium supplemented with 2% sucrose and grown vertically at 22 ± 1 °C under long-day conditions (16 h light/8 h dark) with a light intensity of 100 µmol m^−2^ s^−1^. To assess light sensitivity, 3-day-old dark-grown *abi4* mutants were transferred to light for 12, 24, or 48 h. Whole seedlings were then collected for chlorophyll analysis.

### 4.2. Anthocyanin Determination

Anthocyanin levels were measured using a previously described method [42]. Four-day-old seedlings were incubated overnight at 4 °C in 600 μL of 1% HCl in methanol. The extract was then mixed with an equal volume of chloroform, followed by the addition of 400 μL of water. After centrifugation at 12,000 rpm for 15 min at 25 °C, anthocyanin content was quantified using the absorbance difference (A530 − 0.25A657) and normalized to fresh weight. One unit of anthocyanin was defined as the absorbance value in 1 mL of extraction solution.

### 4.3. Chlorophyll Determination

Chlorophyll content was determined using a previously reported method with minor modifications [43]. Briefly, fresh plant tissue was homogenized in 80% acetone, and the supernatant was collected after centrifugation. Absorbance at 663 and 645 nm was measured using a 722N spectrophotometer (Shanghai Yoke Instrument Co., Ltd., Shanghai, China).

### 4.4. Analysis of Chloroplast Ultrastructure

Cotyledons of seedlings were prepared for transmission electron microscopy (TEM) by fixation in 2.5% glutaraldehyde, followed by post-fixation in 1% osmium tetroxide. Samples were dehydrated through a graded ethanol series (30%, 50%, 70%, and 90%) and embedded in resin. Ultrathin sections were cut using an ultramicrotome, mounted on copper grids, stained with uranyl acetate and lead citrate, and examined using a JEM-2100 TEM instrument (JEOL, Tokyo, Japan).

### 4.5. Gene Expression Analysis

Total RNA was extracted using TRI Reagent and treated with DNase I to eliminate genomic DNA. First-strand cDNA was synthesized using the GoScript™ Reverse Transcription System following the manufacturer’s instructions. Quantitative PCR (qPCR) was performed using a CFX96 Real-Time PCR System (Bio-Rad, Hercules, United States) with Hieff^®^ qPCR SYBR^®^ Green Master Mix (Yeasen, Shanghai, China). The thermal cycling conditions were as follows: initial denaturation at 95 °C for 2 min, followed by 40 cycles of 95 °C for 10 s and 60 °C for 30 s. Gene expression levels were calculated according to the method described in our previous study, with *ACTIN2* used as the internal reference gene [44]. Primer sequences are listed in Table S1.

### 4.6. Identification of Mutants

The single-point mutants (*abi4-1* and *abi4-101*) were identified by direct sequencing of genomic PCR products using gene-specific primers (Table S2). The T-DNA insertion mutants (*abi4-2* and *hy5*) were identified by genomic PCR using specific primers (Figure S1). RT-PCR analysis was conducted with the gene-specific primers for *ABI4* and *HY5* (Figure S2). The *β-ATP* gene was used as an internal control. Sequences of primers used for PCR and RT-PCR are listed in Table S3.

### 4.7. Statistical Analysis

For each experiment, three independent biological replicates were conducted, and the mean ± standard deviation (SD) values were used to present the data. A one-way analysis of variance (ANOVA) was performed on the data using SPSS 25.0 (IBM, Armonk, United States) and by employing Duncan’s test at a significance level of *p* < 0.05. Significant differences are indicated by distinct lowercase letters.

## 5. Conclusions

In conclusion, this study reveals that, beyond its established roles in lateral root development, lipid metabolism, retrograde signaling, and stress response, ABI4 also plays a crucial role in regulating anthocyanin biosynthesis. Our findings demonstrate that ABI4 acts as a negative regulator of anthocyanin accumulation in a HY5-dependent manner. However, whether ABI4 directly binds to the promoters of anthocyanin biosynthetic genes remains to be determined. Future studies should aim to clarify the precise molecular mechanisms through which ABI4 regulates this process.

## Data Availability

The data supporting reported results can be found in the manuscript and Supplemental Materials.

## Supplementary Materials

The following supporting information can be downloaded at: https://www.mdpi.com/article/10.3390/plants14131905/s1, Figure S1: PCR-based identification of T-DNA insertion mutants; Figure S2: Expression analysis of *ABI4* and *HY5* in the T-DNA insertion mutants by RT-PCR; Table S1: Primers used for quantitative real-time PCR in this study; Table S2: Sequences of wild-type, *abi4-1*, and *abi4-101* mutants; Table S3: Primers used for identifying mutants.
