# Supplementary material for: Disruption of ABI4 Enhances Anthocyanin Accumulation in Arabidopsis Seedlings Through HY5-Mediated Light Signaling
Source: Plants (Basel). 2025 Jun 20;14(13):1905. doi: 10.3390/plants14131905 (PMC12251728; doi:10.3390/plants14131905)
Supplement: Supplementary file 1 [file plants-14-01905-s001.zip › Supplementary Figures.pdf]

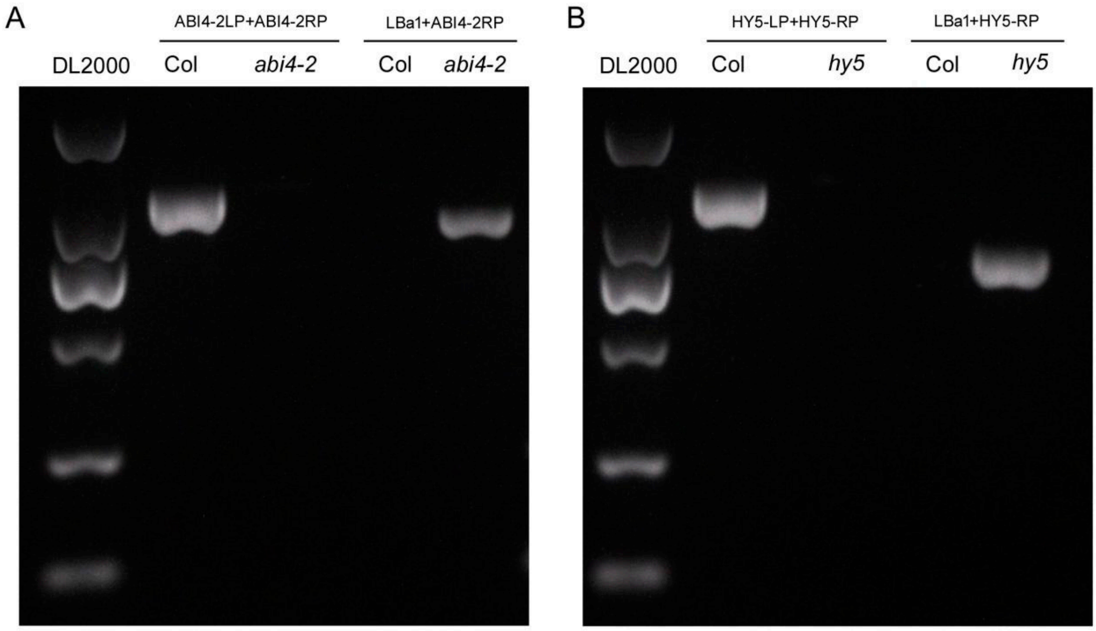

Figure S1. PCR-based identification of T-DNA insertion mutants. DNA was extracted from 4-d-old seedlings and subjected to PCR using specific primers. (**A**) For *ABI4*, primers were designed to amplify the fragment from the *abi4-2* mutant (LBa1+ABI4-2RP) and the fragment from the wild-type (Col) seedlings (ABI4-2LP+ABI4-2RP). (**B**) For *HY5*, primers were designed to amplify the fragment from the *hy5* mutant (LBa1+HY5-RP) and the fragment from the wild-type (Col) seedlings (HY5-LP+HY5-RP).

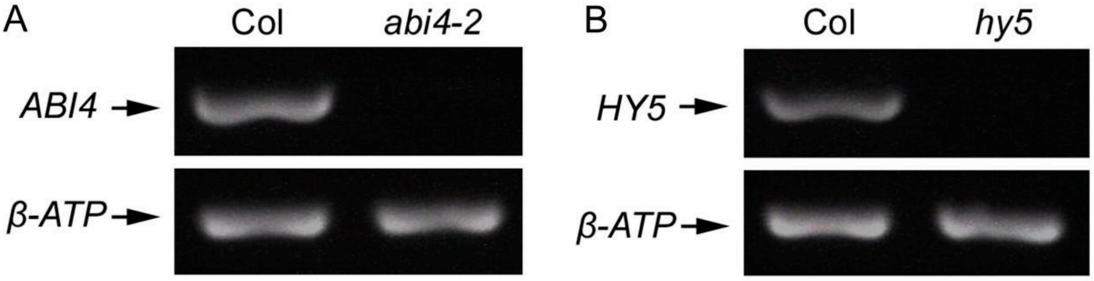

Figure S2. Expression analysis of *ABI4* and *HY5* in the T-DNA insertion mutants by RT-PCR. (**A**) For *ABI4*, primers were designed to amplify the fragment from the wild type (Col) and *abi4-2* mutant (ABI4R-F+ABI4R-R). (**B**) For *HY5*, primers were designed to amplify the fragment from the wild type (Col) and the *hy5* mutant (HY5R-F+HY5R-R). The *β-ATP* gene was used as an internal control (β-ATPR+β-ATPS).
