# Supplementary material for: Disruption of ABI4 Enhances Anthocyanin Accumulation in Arabidopsis Seedlings Through HY5-Mediated Light Signaling
Source: Plants (Basel). 2025 Jun 20;14(13):1905. doi: 10.3390/plants14131905 (PMC12251728; doi:10.3390/plants14131905)
Supplement: Supplementary file 1 [file plants-14-01905-s001.zip › Supplementary Tables.pdf]

Table S1. Primers used for quantitative real-time PCR in this study.

Gene name	Forward primer (5'-3')	Reverse primer (5'-3')
CHS	GGCAAAGAAGCGGCAGTGAAG	GGAAGGACGGAGACCAAGAAG
CHI	TCATGTAGACTCCGTCACGTTTG	TGACAGATAGAGAAGGAACGGCG
F3'H	TTCCTTACCTTCAGGCGGTTATC	CGAGAGTGGTGTTGGTGGATG
DFR	CTTTGTTCGTGCCACCGTTCG	AAAATCCATGGGTGTTGCCAC
LDOX	GTTTGCAGCTTTTCTACGAGGGC	ATGTTGAGCAAAAGTCCGTGGAG
UF3GT	TTGTCAGATCGTTTTGGTTCCGC	TCTTCCTCACTTTCTCACCGATC
UGT75C1	GCTGTTTTGGCGCATTGTGC	TCAGCAAACTGCGGAAACG
LHCB1.1	GGTTTGTGTTTGTGGTGGATGGTA	GTGAACCCAAGAACTGAAAATCCA
CHL27	TCAAGACCGATTACAACCAGACA	CGCTCAAGGAACTCAACGAAG
RBCS	GGTCGCTCCTTTCAACGGACTT	ATTCGGAATCGGTAAGGTCAGG
LHCB4	CAAGTTCTTTGACCCGCTAGG	GATGATGGTGGTGTGGAGTGG
OE23	GTATCTCCTGCTGATGCCGCC	TGGAACCTGCACTTTGAACCC
PSAN	CTGTGATCAAAGCTCAACGCG	TTGGTTTTGCTCCTCTCGAGG
PSBB	CGTTGGGTATATTAGCGGGCC	AGGACGGTTTCAATATTGCCC
PSAA	TCCTGAATGGAGATGTGGGCG	TAAGGCTGCGAAGACCAATGC
PSAB	GTATTACCGCATCCCCAAGGG	GGGTTAGAATGGCAGTTCCGG
ACTIN2	CAAACGAGGGCTGGAACAAGACT	CTGTTGACTACGAGCAGGAGATGG

Table S2. Sequences of wild-type, *abi4-1*, and *abi4-101* mutants.

		Wild-type		Mutants	
	Codon	DNA[a]	Amino acid[b]	DNA[a]	Amino acid[b]
abi4-1	157	GCC	A	ΔCC	Frameshift
abi4-101	193	CAA	Q	TAA	Stop

The mutation sites were identified by direct sequencing of genomic PCR products using gene-specific primers (ABI4D-F+ABI4D-R). [a]DNA sequence of indicated codon; [b]Amino acid specified by indicated codon.

Table S3. Primers used for identifying mutants.

Primer name	Primer sequence (5'-3')
ABI4D-F	AGATCCGAGAGCCACGTAAGCG
ABI4D-R	CCAGACCCATAGAACATACCGG
LBa1	TGGTTCACGTAGTGGGCCATCG
ABI4-2LP	TGAATGCCTTGGAGTGTTTTC
ABI4-2RP	GTGTTGGAATTGTCCCATCTG
ABI4R-F	ATGGACCCTTTAGCTTCCCAAC
ABI4R-R	TTACCGGAACATCAGTGAGCTC
HY5-LP	ATTCCTTCCCAAAATGTCTCG
HY5-RP	ATGCGAGTGAATGACCATTTC
HY5R-F	GGCTGAAGAGGTTGTTGAGGAAC
HY5R-R	ACCACCTCCTCTCTTGTTTCCTG
β-ATPR	GATCATGACATCTCTCGAGG
β-ATPS	TGGTAAGGAGCAAGGAGATC
